# The human immune response to *Toxoplasma*: Autophagy versus cell death

**DOI:** 10.1371/journal.ppat.1006176

**Published:** 2017-03-09

**Authors:** Shruthi Krishnamurthy, Eleni K. Konstantinou, Lucy H. Young, Daniel A. Gold, Jeroen P. J. Saeij

**Affiliations:** 1 Department of Pathology, Microbiology and Immunology, University of California Davis, Davis, California, United States of America; 2 Department of Ophthalmology, Harvard Medical School, Boston, Massachusetts, United States of America; University of Wisconsin Medical School, UNITED STATES

## Introduction

**Fig 1:**
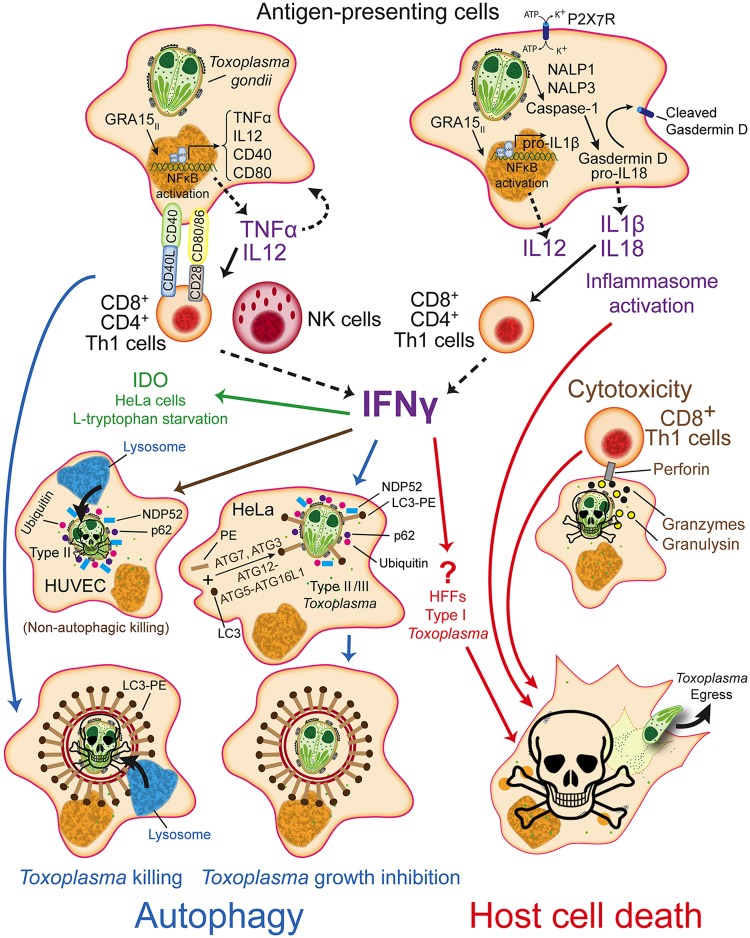
IFNγ dependent and independent toxoplasmacidal pathways in humans. See the main text for explanations.

*Toxoplasma gondii* is an obligate intracellular parasite that can infect a broad range of warm-blooded animals. In humans, it can cause serious disease in immune-compromised individuals or if contracted congenitally. In North America and Europe, strains belonging to the clonal types I, II, and III haplogroups predominate, while in South America a large variety of other “atypical” strains exist. These strains differ enormously in virulence in mice and there is evidence that different strains can also cause variable pathology in humans. In mice, the cytokine interferon gamma (IFNγ) induces multiple toxoplasmacidal mechanisms and therefore plays a crucial role in immunity to *Toxoplasma*. Compared to mice, humans are more resistant to *Toxoplasma*. This is surprising because humans lack the Toll-like receptors 11/12 (TLR11/12) found in mice that bind to the *Toxoplasma* protein profilin and trigger a signaling cascade leading to the production of interleukin 12 (IL12), a key cytokine that stimulates IFNγ production. Humans also lack the multitude of murine immunity-related GTPases (IRGs) that are induced upon IFNγ stimulation and play a crucial role in the destruction of the membrane surrounding the parasitophorous vacuole (PV) in which *Toxoplasma* resides in the host cytoplasm. Thus, the mechanisms involved in the production of IFNγ in humans (Fig 1) differ from those in mice and the pathways that mediate parasite clearance are less well understood (reviewed in [1]). In this review, we focus on two mechanisms of *Toxoplasma* clearance by human cells: autophagy and cell death.

## IFNγ-dependent, noncanonical autophagy-mediated clearance of *Toxoplasma* in humans

Autophagy is a degradation process that clears cytoplasmic material such as misfolded proteins and damaged organelles. Canonical autophagy involves the formation of a double membrane structure, the phagophore, which elongates to engulf cytoplasmic material. During autophagy, cytosolic microtubule-associated protein light chain 3 (LC3), a ubiquitin-like protein, is processed and conjugated to the lipid phosphatidylethanolamine (PE) to form LC3-II on the autophagosome membrane by a set of autophagy-related proteins (ATGs). Ubiquitinylated cargo bound to ubiquitin-binding proteins such as p62 and nuclear dot protein 52 kDa (NDP52) then gets recruited through these proteins’ LC3-interacting regions [2]. The double membrane structure eventually closes to form the autophagosome, which ultimately fuses with lysosomes leading to degradation of the ubiquitinylated cargo.

Noncanonical autophagy requires only a subset of the ATG proteins and these proteins can also be recruited to other membranes besides the phagophore, such as the *Toxoplasma* PV membrane (PVM). For example, in IFNγ-stimulated murine cells, the ATG proteins that mediate the processing, lipidation, and attachment of LC3 to the PVM (ATG7, ATG3, and the ATG12-ATG5-ATG16L1 complex) are needed for the PVM recruitment of two subclasses of IFNγ-inducible GTPases: IRGs and guanylate binding proteins (GBPs). Once these GTPases are recruited to the LC3-tagged PVM, they mediate its vesiculation, resulting in parasite exposure to the cytosol and subsequent parasite and/or host cell death. In contrast to canonical autophagy, lysosome function and the ATG proteins that form the initial phagophore are dispensable [3]. In this sense, LC3 can be viewed as a protein that can tag membranes for subsequent destruction analogous to how ubiquitin tags proteins for destruction [4].

The two human IRGs, immunity-related GTPase family M protein (IRGM; truncated compared to mice) and immunity-related GTPase cinema (IRGC; only expressed in the testes), are not IFNγ-inducible. IRGM is known to play a role in autophagy and host defense to *Mycobacterium tuberculosis* [5], but its role in toxoplasmosis has not been investigated. Humans do express seven IFNγ-inducible GBPs that are involved in IFNγ-dependent clearance of pathogens such as *Chlamydia* and resistance towards certain viruses [6]. However, there is currently no evidence that these human GBPs are involved in IFNγ-mediated restriction of *Toxoplasma*, as they were not observed to localize around the PV of *Toxoplasma* in infected human foreskin fibroblasts (HFFs), and knockdown of *GBP1* and *GBP2* did not change the level of IFNγ-mediated restriction of *Toxoplasma* [7]. Furthermore, complete removal of the *GBP* locus in haploid HAP1 human cancer cells did not affect the IFNγ-induced *Toxoplasma* growth suppression [8]. By contrast, GBP1 was required for growth restriction of the type II but not the type I *Toxoplasma* strain, even in the absence of IFNγ in the human lung epithelial cell line A549 despite its lack of decoration around *Toxoplasma* PVs [9].

In human cells, a role for IFNγ-mediated noncanonical autophagy in clearing *Toxoplasma* infection in a strain and cell type-specific manner has been described. Infection of HeLa cells with the type II and III strains, but not with the type I strain, leads to decoration of PVs with ubiquitin, LC3, p62, and NDP52 (Fig 1). Parasites in PVs that were positive for these autophagy markers had a reduced growth rate but the PVs were never observed to fuse with lysosomes. This noncanonical autophagy was only seen when cells were prestimulated with IFNγ and the strain differences were independent of known *Toxoplasma* mouse-specific virulence factors (polymorphic secreted proteins: ROP18, ROP5, ROP17) that antagonize the IRGs and GBPs [2]. IFNγ-stimulated human umbilical vein endothelial cells (HUVEC) kill type II (but not type I) parasites through another mechanism by which proteins on the PVM get ubiquitinylated in the absence of LC3 deposition, leading to the recruitment of p62 and NDP52 and subsequent endosome/lysosome fusion, PV acidification, and parasite killing, without the involvement of autophagy (Fig 1) [10]. The mechanism for the growth restriction of types II/III in IFNγ-stimulated Hela cells, the identity of the ubiquitinylated proteins on the PVM in IFNγ-stimulated HeLa and HUVEC cells, the ubiquitin ligase that attaches ubiquitin to these proteins, and the *Toxoplasma* protein(s) that determine the strain differences in these processes are unknown. In IFNγ-stimulated HFFs, LC3 was also not recruited to the PVM of type I parasites [7]. Instead, type I infection induced rapid host cell death, which likely restricted parasite growth. In IFNγ-stimulated HeLa cells, type I parasite growth is also restricted, but this is dependent on the IFNγ-induced enzyme Indoleamine-2,3-dioxygenase (IDO), which degrades L-tryptophan, an amino acid for which *Toxoplasma* is auxotrophic (Fig 1; for a summary of cell type and species differences in IDO-mediated restriction of *Toxoplasma* see Table 2 in [11]). Because nutrient starvation can induce autophagy, it is possible that IFNγ-induced L-tryptophan starvation triggers *Toxoplasma* clearance via noncanonical, ubiquitin-mediated autophagy.

## IFNγ-independent, autophagy-mediated clearance of *Toxoplasma* in humans

The costimulatory protein cluster of differentiation 40 (CD40) is expressed on the surface of antigen presenting cells (APCs), such as macrophages, and on some nonhematopoietic cells. In human and murine cells, CD40 interacts with its CD40L ligand, which is expressed on the surface of T cells, leading to T cell production of IFNγ, which is only partially dependent on macrophage production of IL12 (Fig 1). CD40–CD40L interactions lead to autocrine production of Tumor Necrosis Factor (TNFα), which is needed for CD40–CD40L-signaling to induce autophagy-mediated clearing of *Toxoplasma*-containing PVs by fusion of LC3-decorated PVs with lysosomes (Fig 1) [12]. The importance of CD40–CD40L-mediated *Toxoplasma* destruction in humans is supported by the fact that patients with HyperIgM syndrome—where the communication between T cells and APCs is impaired due to defective CD40L expression—are more susceptible to *Toxoplasma* [13]. Furthermore, CD4 T cells from HIV patients, which are defective in CD40L induction, display impaired cell-mediated immunity against *Toxoplasma* infection [13]. A more dominant role for CD40–CD40L-mediated immunity in humans compared to rodents may explain why IL12/IFNγ pathway-deficient human patients are not more susceptible to *Toxoplasma*, while mice deficient in IL12/IFNγ pathway genes are extremely susceptible to *Toxoplasma* [14]. Indeed, in vitro studies revealed that residual IFNγ-responsiveness in patients with partial IFNγ receptor 1 deficiency in the presence of TNFα is sufficient to inhibit *Toxoplasma* but not *Salmonella* proliferation, possibly explaining why these patients are more susceptible to bacteria but not to *Toxoplasma* [15]. It is therefore likely that in humans, contrary to mice, CD40–CD40L-dependent toxoplasmacidal mechanisms can compensate for the lack of IL12 and IFNγ. CD40(-/-) mice control acute infection but are susceptible to cerebral and ocular toxoplasmosis [13], likely because CD40–CD40L interactions have a role in mediating long-term CD8 T cell immunity and in preventing CD8 T cell exhaustion (reviewed in [16]).

## Role of host cell death in clearance of *Toxoplasma* in humans

Host cell death inhibits *Toxoplasma* proliferation, which requires a host cell to replicate and survive (Fig 1). Thus, it is not surprising that *Toxoplasma* strongly inhibits apoptosis, possibly through inhibition of caspases 3/7/8, which are proteolytic proenzymes that can trigger cell death upon activation (reviewed in [17]). However, inhibition of caspase-8 sensitizes cells towards necroptosis, a programmed form of inflammatory cell death, because caspase-8 is an inhibitor of receptor-interacting serine/threonine protein kinase (RIPK3), a key mediator of necroptosis [18]. It is therefore likely that upon IFNγ, TNFα, or TLR3 activation, which can all activate RIPK3, *Toxoplasma*-infected cells can die from necroptosis. HFFs normally support the growth of *Toxoplasma*; however, IFNγ-stimulated HFFs rapidly die through an unknown mechanism upon infection with the type I strain, triggering premature parasite egress [7]. Human cytotoxic T cells can kill *Toxoplasma*-infected target cells by secreting pore-forming perforins into the infected cells and subsequently secreting granzymes through these pores, which can kill the infected cell by activating caspases. Human, but not rodent, T cells also secrete the antimicrobial peptide granulysin through these pores, which can destroy the *Toxoplasma* PVM, allowing granzymes to enter the parasite where they kill the parasite through generation of reactive oxygen species (Fig 1)[19].

Intracellular recognition of pathogens is mediated across many cell types by the family of cytoplasmic pattern recognition receptors (PRRs) called nucleotide-binding oligomerization domain-like receptors (NLRs). Upon activation by pathogen-associated molecular patterns (PAMPS), they form a large protein complex called the inflammasome together with caspase-1. Caspase-1 regulates the maturation of the pro-inflammatory cytokines IL1β and IL18 and cleaves gasdermin D, which subsequently can form pores in the cell membrane, thereby inducing a form of cell death called pyroptosis. For example, macrophages from Lewis rats are naturally resistant to *Toxoplasma* infection, likely because they undergo rapid NLRP1-mediated pyroptosis upon infection, thereby eliminating its replication niche. In humans, the *NALP1* gene has been associated with susceptibility to congenital toxoplasmosis. Knockdown of *NALP1* in a human monocyte cell line resulted in lower levels of IL1β, IL18, and IL12, enhanced parasite replication, and subsequent accelerated monocyte cell death. However, unlike Lewis rat macrophages, no role for NALP1 in pyroptosis was described in humans, and the increased cell death was likely due to increased parasite burden (reviewed in [20]). Whether other *NALP1* alleles induce pyroptosis in human cells upon *Toxoplasma* infection remains to be investigated. Similarly, the *ALOX12* gene, which encodes the enzyme arachidonate 12-lipoxygenase, has allelic variants associated with toxoplasmosis and *ALOX12* knockdown prevents *Toxoplasma* tachyzoites from proliferating likely by increasing host cell death [21].

One final example of cell death-mediated resistance involves purinergic receptors (P2X7R) found on the surface of macrophages, which are activated by extracellular ATP and can be up-regulated synergistically by IFNγ and TNFα (Fig 1). Human polymorphisms in the *P2X7R* gene influence susceptibility to toxoplasmosis and macrophages from people with *P2X7R* loss-of-function alleles have a reduced ability to inhibit *Toxoplasma* growth upon ATP stimulation likely because of a defect in host cell death upon ATP stimulation (reviewed in [22]). Clearly, more studies are required to delineate the role of different human inflammasomes in toxoplasmosis.

## Conclusion

Different cell lines from the same species or similar cell types from distinct species can vary enormously in the expression of indoleamine 2,3-dioxygenase [11], inducible nitric oxide synthase, RIPK3 [18], NLRs, and IRGs, likely explaining cell type and species differences in response to *Toxoplasma*. Furthermore, different *Toxoplasma* strains not only vary in their ability to block the variety of toxoplasmacidal mechanisms but they also differ in the activation of host signaling pathways that can lead to differences in expression of host toxoplasmacidal effectors. For example, type II parasites activate the NFκB transcription factor (predominantly the p50/p65 heterodimer) via the secreted protein GRA15. This eventually leads to enhanced secretion of IL1β and higher expression of CD40 by human monocytes (reviewed in [17]). Because IL1β can enhance the IL12-mediated stimulation of IFNγ production [23] and CD40–CD40L interaction can activate *Toxoplasma* destruction through autophagy, it is likely that GRA15 plays a role in human toxoplasmosis. The timing and cellular context for IFNγ-dependent and -independent toxoplasmacidal mechanisms in humans remain unclear and warrant further research.
